# A Narrative Review of Injury Incidence, Location, and Injury Factor of Elite Athletes in Snowsport Events

**DOI:** 10.3389/fphys.2020.589983

**Published:** 2021-01-08

**Authors:** Yongxin Xu, Chenhao Yang, Yang Yang, Xini Zhang, Shen Zhang, Mingwen Zhang, Li Liu, Weijie Fu

**Affiliations:** ^1^School of Kinesiology, Shanghai University of Sport, Shanghai, China; ^2^Beijing Institute of Fashion Technology, Beijing, China; ^3^Institute for Frontier Materials, Deakin University, Geelong, VIC, Australia

**Keywords:** Winter Olympics, World Cup, snowsport, injury surveillance study, elite athlete

## Abstract

Snowsport athletes face a high injury risk both during training and in competitions. Reducing injury incidence is crucial for athletes to achieve breakthroughs. This narrative review aimed to summarize and analyze injury data of elite athletes in snowsports and provide references for injury prevention and health security for these athletes and their coaches. A total of 39 studies that investigated snowsport injury were analyzed in the present study. On the basis of injury data of elite athletes in snowsports events, this narrative review focused on four aspects, namely, injury incidence, severity, location and causes. The findings of this review were as follows. (1) The highest injury incidence was recorded in freestyle skiing, followed by alpine skiing and snowboarding, the majority of which were moderate and severe injuries. (2) The proportion of injury in competitions and during training was similar. However, more injuries occurred in official training during the Winter Olympic Games; by contrast, injury proportion was higher in competitions during World Cup/World Championships. (3) The most commonly and severely injured body parts were the knees (29.9%), head and face (12.1%), shoulders and clavicula (10.5%), and lower back (8.9%). The most common injury types were joint and ligament injury (41.5%), fracture and bone stress (24.4%), concussion (11.1%), and muscle/tendon injury (10.7%). (4) The main causes of snowsport injury were collisions, falls, and non-contact injuries. Snowsport injury was also influenced by the skill level of the athletes, gender, course setup and equipment. Future studies should further explore the influence of event characteristics and intrinsic and extrinsic risk factors on snowsport injury. An injury or trauma reconstruction may be developed to predict athletic injuries and provide effective prevention strategies.

## Introduction

Professional snowsport racing is characterized by high skiing speed, spectacular jumps and complex skills under cold ambient conditions (Gilgien et al., 2014; Wijdicks et al., 2014; Racinais et al., 2017). However, the spectacular falls, crashes, and injuries of athletes that we regularly see during training and in competitions demonstrate that these elite athletes are highly prone to musculoskeletal injury that may even lead to disability or death (Engebretsen et al., 2010; Weber et al., 2016; Supej et al., 2017; Watanabe et al., 2019). Thus, a systematic injury surveillance is needed for the effective protection of the health of athletes (Soligard et al., 2019). On the basis of this premise, the “sequence of prevention” model (van Mechelen et al., 1992) provides a common framework for injury surveillance and prevention. Injury epidemiology and etiology are considered as the first and second steps for injury prevention, respectively.

Epidemiological studies based on this conceptual model provide information that allows evidence-based decision-making concerning risk levels and the efficacy of preventive and therapeutic interventions (Fuller and Drawer, 2004). These interventions can be described by injury incidence and severity. Furthermore, epidemiological studies should identify the etiology of sports injury (van Mechelen et al., 1992). This inquiry should include obtaining information on how injuries happen (injury mechanisms) and why an athlete may be at risk in a given situation (injury factors). Although an injury may appear to be caused by a single inciting event, it may actually be result of a complex interaction between intrinsic (personal) and extrinsic (environmental) risk factors (van Mechelen et al., 1992; Bahr and Krosshaug, 2005).

The “sequence of prevention” model has become an important reference of injury risk management by the International Ski Federation (FIS). The FIS has published a series of prospective injury reports and has conducted retrospective interviews to identify and prevent injury among elite athletes in snowsports. These studies covered injury epidemiology (Engebretsen et al., 2010; Ruedl et al., 2012; Soligard et al., 2015, 2019; Steffen et al., 2017; Watanabe et al., 2019), mechanism (Bere et al., 2011, 2013; Steenstrup et al., 2018) and causes and factors (Spörri et al., 2012). However, these previous studies were often limited to only one or a few events and disciplines. Only a handful of studies have summarized injury epidemiology and etiology among elite athletes of snowsports (Flørenes et al., 2012; Oslo Sports Trauma Research Center, 2019).

This review aimed to summarize and analyze the data of injury surveillance studies concerning elite athletes in major snowsport events (Winter Olympic Games, Youth Winter Olympic Games, World Cups) from the 2006 to the 2019 season. This review described injury incidence, severity, location and type and identified the factors and causes of these injuries with the long-term goal of preventing injuries.

## Literature Search Methodology

**FIGURE 1:**
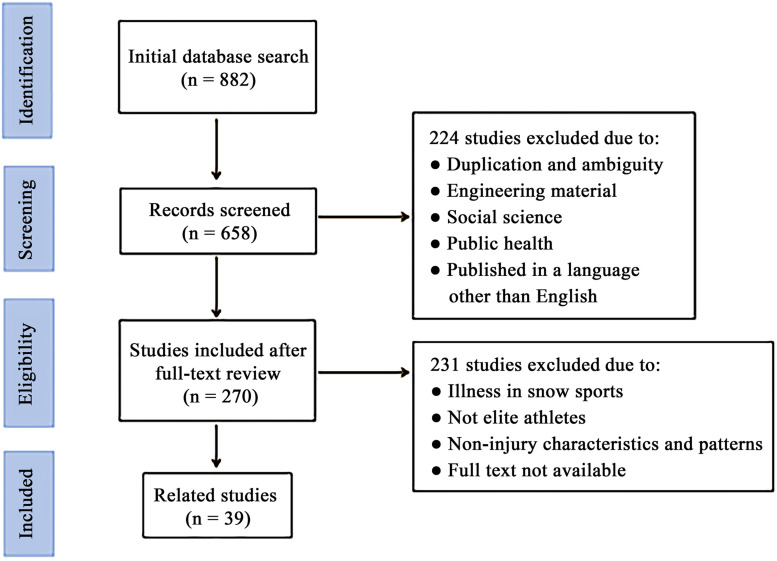
Flow diagram of the literature selection process and the number of articles (*n*) after each stage.

Relevant studies were identified by searching the databases of PubMed and Web of Science from study inception until March 2020. Inclusion criteria were as follows: peer-reviewed, available in full text and published in English. The search terms used were “Winter Olympic, winter sports, alpine skiing, cross-country skiing, freestyle skiing, snowboarding” AND “injury, trauma, injury prevention.” Moreover, the authors described injury characteristics and injury patterns and about elite athletes in snowsports. Exclusion criteria were as follows: had duplicate and ambiguous literature; about engineering materials, social science and public health; and about illness in snowsports. A flow diagram describing the detailed search strategy, exclusion criteria and article selection process is shown in Figure 1.

## Injury Incidence and Severity

### Injury Incidence

To monitor injury patterns and trends in different FIS disciplines and events, the FIS Injury Surveillance System (FIS–ISS) arranged major events of snowsports (i.e., Winter Olympic Games, World Cups and World Championships) to obtain a complete background data for in-depth studies of injuries. It showed that the risk for injury in alpine skiing, freestyle skiing and snowboarding was higher (Flørenes et al., 2012). Analyzing injury incidence from 13 seasons (2006–2019), the injury incidence in snowboarding, freestyle skiing and alpine skiing can reach about 20.7–68.1 injuries per 100 athletes, 25.4–53.0 injuries per 100 athletes, and 23.5–45.8 injuries per 100 athletes, respectively (Oslo Sports Trauma Research Center, 2019). Compared with other disciplines monitored by FIS–ISS, the injury rate was higher in snowboard cross (11.9 injuries per 1,000 runs) and halfpipe (6.3 injuries per 1,000 runs) (Major et al., 2014), aerials (19.2 injuries per 1,000 runs), halfpipe skiing (23.9 injuries per 1,000 runs), ski cross (18.5 injuries per 1,000 runs) (Flørenes et al., 2010), and downhill in alpine skiing (17.2 injuries per 1,000 runs) (Flørenes et al., 2009). Furthermore, according to the injury data in alpine skiing from three World Cup seasons (2012–2015) (Haaland et al., 2016), the disciplines with the highest injury incidence were downhill skiing (18.1 injuries per 1,000 runs), followed by Super-G (11.1 injuries per 1,000 runs), giant slalom (9.5 injuries per 1,000 runs), and slalom (4.0 injuries per 1,000 runs).

**FIGURE 2:**
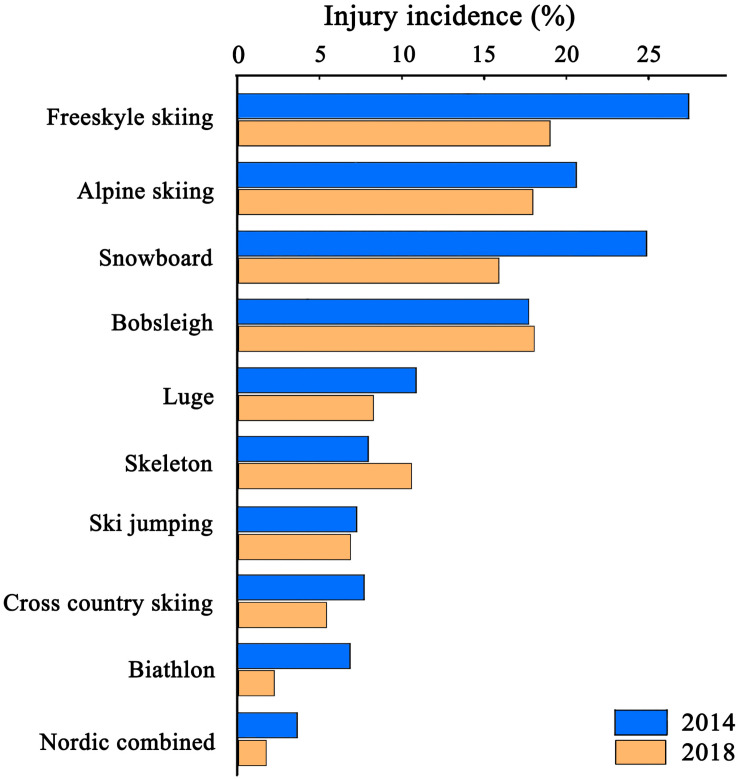
Injury incidence in snowsports during the 2014 Sochi and 2018 PyeongChang WOGs. Blue bars indicate injury incidence in the 2014 Sochi WOG, whereas orange bars denote injury incidence in the 2018 PyeongChang WOG.

Specifically, we take the last two Winter Olympic Games (WOG), i.e., 2014 Sochi and 2018 PyeongChang, for examples. The previous studies summarized injury incidence in snowsports in the two competitions (Figure 2; Soligard et al., 2015, 2019). They recorded higher injury incidence (>15%) in freestyle skiing, alpine skiing and snowboarding than in other events. By contrast, they reported lower injury incidence (<10%) in Nordic skiing (biathlon, cross-country skiing, Nordic combined and ski jumping). Compared with that in the 2014 Sochi WOG, the injury incidence of each sport in the 2018 PyeongChang WOG was lower, except for bobsleigh and skeleton events. A continuous updating of equipment may contribute to the reduction in injury incidence. For instance, skis used in alpine skiing that less shaped and with a greater sidecut radius reduce the self-steering effect (the ski turns by itself if it is edged and loaded) and contributes to less aggressive ski–snow interaction (Kröll et al., 2016a; Spörri et al., 2016). Moreover, a less-shaped ski can increase the turning radius and reduce ground reaction force and kinetic energy while turning (Spörri et al., 2015; Kröll et al., 2016b; (a). Longer skis can increase user comfort and enhance predictability at high speeds, and skis with a reduced profile width can help athletes get off the edge more easily while carving (Spörri et al., 2012). Thus, less-shaped and longer skis with a reduced profile width have been proved to have a positive effect on injury prevention (Kröll et al., 2016a,b; Spörri et al., 2016).

**FIGURE 3:**
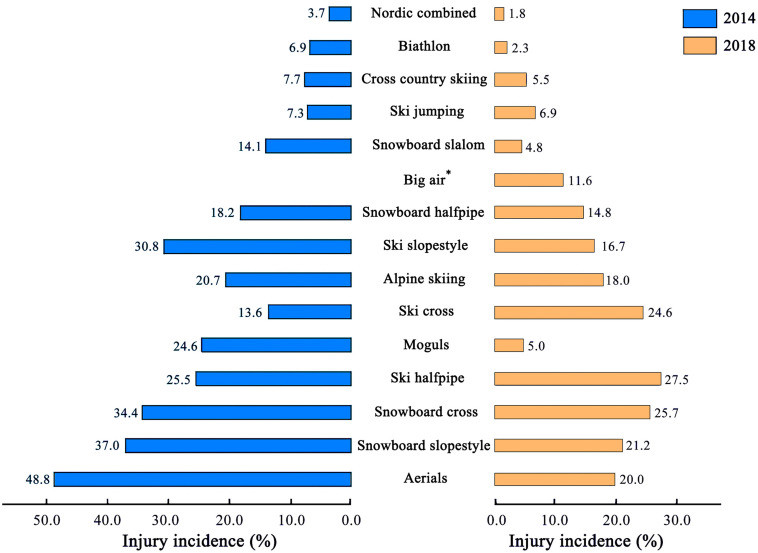
Injury incidence in each discipline during the 2014 Sochi and 2018 PyeongChang WOGs. Blue bars indicate injury incidence in 2014 Sochi WOG, whereas orange bars denote injury incidence in 2018 PyeongChang WOG. ^∗^Big air was not included in the 2014 Sochi WOG.

Figure 3 shows the injury incidence in each discipline. Compared with that in the 2014 Sochi WOG, the injury incidence in the 2018 PyeongChang WOG was lower in biathlon (2 vs. 7%), aerials (20 vs. 49%), and moguls (5 vs. 25%) but higher in ski cross (24.6 vs. 13.6%) and ski halfpipe (27.5 vs. 25.5%) (Soligard et al., 2019). Changes in injury incidence can be a consequence of changes in the composition of Olympic Games program, environmental factors, course design, competition rules or changes in equipment (Soligard et al., 2019). However, owing to the long interval between two Olympic Games (4 years), injury incidence in WOG might be dominated by only a few factors and with contingency. Also, the difference of injury incidence might simply due to a natural variability of athletes’ exposure to risk, and emphasize the value of ongoing surveillance to monitor injury trends over time (Soligard et al., 2015, 2019).

### Injury Proportion in Competitions and During Training

Quantifying the proportion of injuries in competitions and during training is essential for developing injury prevention strategies. On the basis of data on injury proportion through all 13 seasons (2006–2019), injury proportion in snowsports in competitions (52.2%) and during training (47.8%) was fairly even (Oslo Sports Trauma Research Center, 2019), but the trend in different events was slightly different. More injuries occurred during training than in WOG competitions. The proportion of snowsport injury during training for the 2010 Vancouver, 2014 Sochi and 2018 PyeongChang WOGs was 53.4, 66.5, and 56.1%, respectively, whereas the proportion in competitions was 41.5, 30.6 and 42.3%, respectively (Engebretsen et al., 2010; Soligard et al., 2015, 2019). In the 2016 Lillehammer Youth Winter Olympic Games (YWOG), 57.4% of snowsport injuries occurred during training (Steffen et al., 2017). However, in the 2012 Innsbruck YWOG, injuries that occurred in competitions accounted for nearly 60% (Ruedl et al., 2012). A possible reason might be that the training conditions were less competition-specific during this sports event, and the athletes probably had difficulty adapting to a more intense competition. Gradual increases in training intensity to competition level may potentially help reducing ensuing injury incidence in competitions (Ruedl et al., 2012). However, in the World Cup or world championships held annually, more injuries occurred in competitions than during training. In the six seasons (2006–2012) of the alpine skiing World Cup, a total of 577 athletes were injured; 308 in competitions and 269 during trainings (Bere et al., 2014). In addition, a total of 291 acute injuries were recorded in three World Cup freestyle skiing winter seasons (2006–2009). Of the 291 injuries, 74 (25.4%) occurred during official training, whereas 59 (20.3%) injuries transpired from regular training on snow. Four injuries (1.4%) occurred during basic training (not on snow), whereas injuries that happened during trainings accounted for 47.1% (Flørenes et al., 2010). The proportion of injuries during trainings and in competitions might be result of the interval between the two events. WOG is held every 4 years. The time spent training and preparing is longer than in competitions. Accordingly, more injuries occurred during trainings for WOG. By contrast, in events held annually, such as the World Cup and World Championships, athletes may sustain more injuries in competitions than during trainings. On the basis of event interval and characteristics, specific injury prevention strategies for different events are needed to minimize injury incidence.

### Injury Severity

Information on injury severity is essential for setting targets for preventive strategies. Uniform definition of terms are also important in snowsport injury surveillance to ensure the comparability of research data (van Mechelen, 1997). Injury severity is classified according to the duration of absence from training and competition (Flørenes et al., 2012; Oslo Sports Trauma Research Center, 2019; Soligard et al., 2019). The International Olympic Committee injury and illness surveillance system defines severe injuries as injuries estimated to lead to absence from training and competition for more than 1 week (Engebretsen et al., 2010; Soligard et al., 2015, 2019). The FIS–ISS classifies injury severity as slight (no absence), minimal (1–3 days), mild (4–7 days), moderate (8–28 days), and severe (>28 days) (Oslo Sports Trauma Research Center, 2019).

**FIGURE 4:**
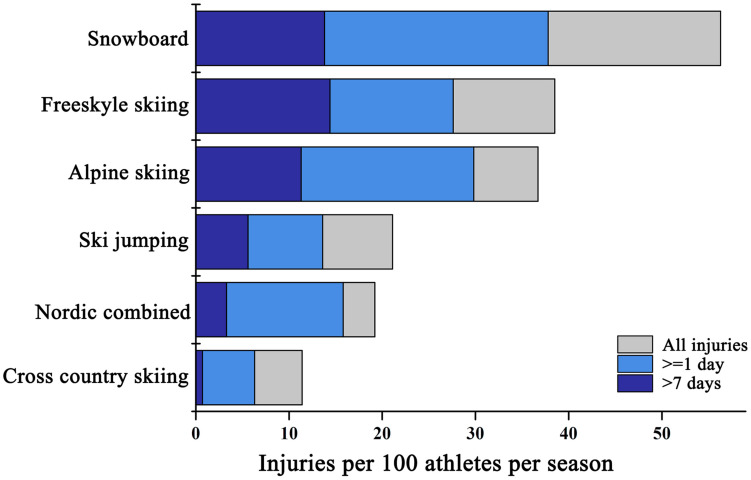
Injuries per 100 athletes per season during the 2006–2007 and 2007–2008 World Cup seasons in alpine skiing, freestyle skiing, snowboarding, ski jumping, Nordic combined and cross-country skiing.

In world-class snowsport events, injuries leading to absence from training and competition represent a substantial proportion of all injuries. From 13 seasons (2006–2019), the incidence of injuries leading to absence in alpine skiing, freestyle skiing and snowboarding accounted for 83.6, 80.8, and 78.4%, respectively (Oslo Sports Trauma Research Center, 2019). In contrast, during the 2014–2019 seasons, the proportion of time-loss injury incidence in ski jumping was lower (69.7%) (Oslo Sports Trauma Research Center, 2019). Of the time-loss injuries, the majority was moderate (time loss 8–28 days) and severe (time loss >28 days) injuries, reported over 50% (Oslo Sports Trauma Research Center, 2019). In 2014 Sochi and 2018 PyeongChang WOGs, the injuries resulted in absence for at least 1 day accounted for 44.6 and 45.0%, respectively. Of these, the severe injuries (time loss >7 days) accounted for 56.5 and 49.4%, respectively (Soligard et al., 2015, 2019). Athletes in 2018 PyeongChang WOG incurred fewer severe injuries. International Olympic Committee employed new algorithms to plan the course design of safe but attractive jumps in certain disciplines. It may provide better protection for athletes (Soligard et al., 2015, 2019). More researches are needed to understand the mechanisms and the situations of time-loss and severe injuries. In the 2016 Lillehammer YWOG, 65.7% of the injuries led to absence from training or competition, whereas 25.0% of all injuries resulted in 1–7 days of absence from the sport. During the 10 days of competition, 10 serious injuries (9.3% of all injuries) were registered with an estimated absence from sport for >7 days (Steffen et al., 2017). The estimated number of injuries per 100 athletes per season during the 2006–2007 and 2007–2008 World Cup seasons is shown in Figure 4 (Flørenes et al., 2012). In terms of severe injuries (time loss >28 days), injury incidence was higher in alpine skiing, freestyle skiing and snowboarding than in ski jumping, Nordic combined and cross-country skiing. Moreover, the rate of severe injuries was higher in ski jumping and Nordic combined than in cross-country skiing (Flørenes et al., 2012). Considering the high speed and vertical drop, jumps and turning around the gate, the high frequency of time-loss injuries and severe injuries may not come as a surprise in snowsports. Future researches are needed to understand the trends and mechanism and develop the appropriate preventive method (Flørenes et al., 2009, 2010; Major et al., 2014).

## Injury Location, Type and Factors

### Injury Location and Type

The incidence of injury location in FIS disciplines reported throughout 13 seasons (2006–2019), expressed as injured body, is shown in Table 1 (Oslo Sports Trauma Research Center, 2019). In world-class snowsport events, the most commonly injured body parts were the knees (29.9%), head and face (12.1%), shoulders and clavicula (10.5%), and lower back (8.9%) (Flørenes et al., 2009, 2010, 2012; Major et al., 2014).

**TABLE 1:** Incidence of injury location (%) expressed as body part injured in various FIS disciplines throughout 13 seasons (2006–2019).

**Injured site**	**Alpine skiing World Cup**	Alpine skiing European Cup*	**Freestyle skiing**	**Snow-boarding**	**Ski jumping^#^**	**Total**
Knees	**41.3**	**36.0**	**32.1**	**16.1**	**41.6**	**29.9**
Head/face	**9.4**	**8.8**	**14.2**	**13.5**	**9.0**	**12.1**
Shoulders, clavicula	**6.1**	**5.0**	**12.0**	**14.3**	**3.4**	**10.5**
Lower back, pelvis, sacrum	**9.2**	**8.4**	**7.0**	**10.7**	**9.0**	**8.9**
Hands, fingers	9.7	13.4	6.3	5.6	0.0	7.3
Ankles	3.8	7.1	5.3	11.2	14.6	7.1
Lower legs, Achilles tendon	9.0	9.6	3.7	2.9	3.4	5.2
Chest (Sternum, ribs, upper back)	1.7	1.7	4.0	4.7	1.1	3.4
Hip/groin	2.1	1.7	4.5	3.3	9.0	3.4
Feet/heels/toes	1.4	2.1	2.3	5.3	1.1	3.0
Wrists	1.2	2.9	2.2	4.6	3.4	2.8
Elbows	0.6	0.8	1.8	2.2	1.1	1.5
Thighs	2.1	1.3	1.4	1.0	0.0	1.5
Neck/cervical spine	0.7	0	1.4	1.6	1.1	1.2
Forearms	0.5	0.4	0.6	1.3	1.1	0.8
Upper arms	0.6	0.4	0.5	1.0	0.0	0.7
Abdomen	0.5	0.4	0.5	0.7	0.0	0.6
Other body parts	0.1	0	0.2	0	1.1	0.1
Total	100.0	100.0	100.0	100.0	100.0	100.0

* Alpine skiing European Cup includes data from six seasons (2013–2019). ^#^Ski jumping includes data from five seasons only (2014–2019). Bold fonts indicate the most common injury types.

**FIGURE 5:**
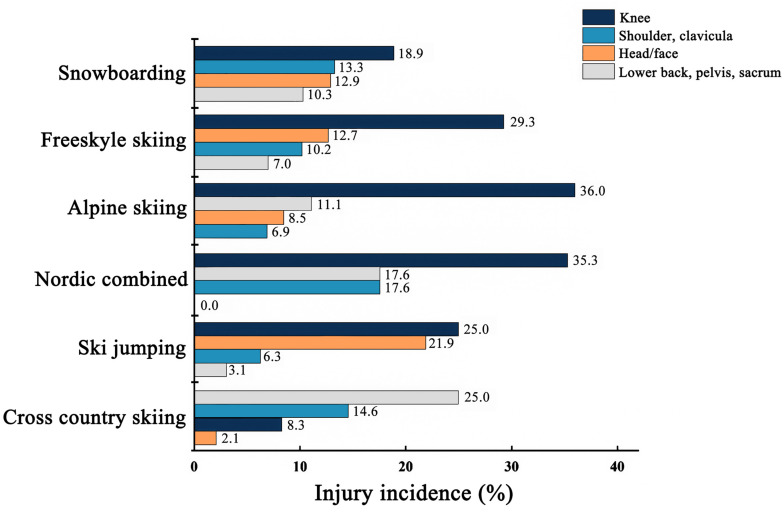
Injury incidence of the commonly injured body parts in different disciplines during the 2006–2007 and 2007–2008 World Cup seasons.

Injury incidence of the commonly injured body parts in each discipline in the 2006–2007 and 2007–2008 seasons is shown in Figure 5 (Flørenes et al., 2012). The most commonly injured body part in cross-country skiing was the lower back (25.0%). By contrast, the highest injury incidence was recorded in the knees in other disciplines (18.9–36.0%). Repetitive back loading in cross-country skiing does not increase the injury risk of the lower back, but the long training hours (>550 h a year) is a risk factor for lower back pain (Foss et al., 2012). In addition, the sagittal configuration of the spine changes in elite adolescent cross-country skiers after 5 years of intensive engagement in the sport (a training volume of 11.7 ± 1.4 h/week). An increase in thoracic kyphosis has been observed in cross-country skiers that may eventually develop to hyperkyphosis over time (Alricsson and Werner, 2006). A strong correlation between lower back pain and an increase in kyphosis of the thoracic spine (*p* = 0.035) was also found in young elite cross-country skiers (Alricsson and Werner, 2006). Thus, cross-country skiing, as an endurance sport that covers long distances that demands large training volume and involves intensive competition, could contribute to a high injury incidence of lower back injuries (Alricsson and Werner, 2006; Foss et al., 2012).

Matsumoto et al. (2002) conducted a prospective survey and found that the injury incidence of upper extremities in snowboarding is almost twice of that in alpine skiing, accounting for nearly 50% of all injuries. Falls and crashes in training and competition are the main causes of upper extremity injuries in snowboarding (Torjussen and Bahr, 2005; Wijdicks et al., 2014). However, the snowboarders included in the present review were at elite level. Their injuries were not strongly associated with an isolated fall. Injuries in lower extremities among elite athletes associated with strong impact caused by jumping and aerial maneuvers have become more traumatic and prevalent (Wijdicks et al., 2014). Errors in take-off result in jumps that are too high and far and end with a “flat landing” (Davies et al., 2009; Bakken et al., 2011; Kim et al., 2012). A flat landing is described as a landing that occur on a horizontal surface out of the transition slope, thus it cannot provide a graded change from a steep inclination to a horizontal surface. The ground reaction force is more perpendicular to the snowboard and in line with the legs in a flat landing. Thus, a higher force is applied that directly compresses the joints. This condition does not provide the legs sufficient time to absorb the impact. Moreover, the time to decelerate is shorter, resulting in a higher impulse on the leg. This type of landing also increases the eccentric contraction of the quadriceps, thereby increasing the loading to the anterior cruciate ligament (ACL) (Davies et al., 2009; Boden et al., 2010) and compounding the risk of injuries to lower extremities and knees (Wijdicks et al., 2014).

In terms of injury location and severity, the knees, shoulders, head/face and lower back were the most commonly injured body parts, the majority of which were severe. Table 2 shows the total proportion of moderate (time loss 7–28 days) and severe (time loss > 28 days) injuries in alpine skiing, freestyle skiing and snowboarding throughout the 13 seasons (2006–2019) (Oslo Sports Trauma Research Center, 2019). The proportion of moderate and severe injuries (time loss > 7 days) accounted for 38.8–80.0% in the aforementioned body parts. By contrast, the body parts with a lower injury incidence were the upper arms (0.4–1.0%), forearms (0.4–1.3%), lower leg/Achilles tendon (2.9–9.6%), ankles (3.8–11.2%), and feet (1.4–5.3%), with the proportion of moderate to severe injuries accounting for over 50%. The body parts with low injury incidence may also potentially lead to long absence from the sport. An injury may prevent an athlete from fulfilling his/her potential and achieve breakthroughs. Therefore, stakeholders and researchers should also focus on these body parts.

**TABLE 2:** Total proportion (%) of moderate and severe injuries (time loss >7 days) reported in alpine skiing World Cup, freestyle skiing and snowboarding throughout the 13 seasons (2006–2019).

**Body part injured**	**Alpine skiing World Cup**	**Freestyle skiing**	**Snowboarding**
Knees	**80.0**	**80.8**	**74.2**
Head/face	**61.8**	**55.4**	**45.0**
Shoulders, clavicula	**45.5**	**64.5**	**40.1**
Lower back, pelvis, sacrum	**43.0**	**38.8**	**46.0**
Hands, fingers	30.5	29.1	28.4
Ankles	59.5	55.2	66.5
Lower legs, Achilles tendon	76.5	53.2	73.0
Chest (Sternum, ribs, upper back)	44.4	52.0	51.7
Hip/groin	47.8	50.9	45.2
Feet/heels/toes	92.9	51.7	55.2
Wrists	23.1	50.0	33.8
Elbows	50.0	47.8	53.6
Thighs	69.5	38.9	38.5
Neck/cervical spine	37.5	33.4	40.0
Forearms	60.0	50.0	70.7
Upper arms	71.5	100.0	92.3
Abdomen	80.0	16.6	44.4
Other body parts	1.0	0.0	0.0

*Injury location is expressed as body part injured. The bold fonts represent the proportion of moderate and severe injuries in the most commonly injured body part.*

Table 3 shows the percentage of each injury type in various FIS disciplines reported throughout the 13 seasons (2006–2019) (Oslo Sports Trauma Research Center, 2019). The most common injury types were joint and ligament injuries (41.5%) and fractures and bone stress (24.4%). In alpine skiing, freestyle skiing and ski jumping, nearly half (44.8–49.4%) of the injuries recorded were joint and ligament injuries. Knee ligament injuries were the most common injury type in snowsports (55.6–67.9%). Among these injuries, ACL injuries were the most frequently diagnosis with high severity (Flørenes et al., 2009, 2010; Soligard et al., 2019). In snowsports, spectacular jumps at high speeds are performed on snow surfaces, and technical errors in take-off and landing phases result in falls, collisions and non-contact injuries. In these situations, the force of high impact directed to the joint increases the risk of knee injuries (Major et al., 2014; Soligard et al., 2019).

**TABLE 3:** Percentages of injury types in various FIS disciplines reported throughout the 13 seasons (2006–2019).

**Injury type**	**Alpine skiing World Cup**	Alpine skiing European Cup*	**Freestyle skiing**	**Snowboarding**	**Ski jumping^#^**	**Total**
Joints/ligaments	**47.1**	**44.8**	**43.4**	**33.8**	**49.4**	**41.5**
Fractures/bone stress	**22.9**	**31.8**	**22.5**	**27.0**	**12.4**	**24.4**
Nervous system/concussion	**8.2**	**8.8**	**12.8**	**12.7**	**6.8**	**11.1**
Muscles/tendons	**10.9**	**9.2**	**9.4**	**11.5**	**19.1**	**10.7**
Contusion	6.2	4.2	8.7	11.7	6.8	8.7
Skin/lacerations	2.8	0.8	0.8	0.9	2.2	1.4
Other injuries	1.2	0.4	1.7	1.6	2.2	1.5
Information missing	0.7	0.0	0.7	0.8	1.1	0.7
Total	100.0	100.0	100.0	100.0	100.0	100.0

* Alpine skiing European Cup includes data from six seasons (2013–19). ^#^Ski jumping includes data from five seasons only (2014–2019). The bold fonts represent the percentages in the most commonly injury types.

### Injury Factors

A comprehensive perspective concerning injury factor is needed in developing effective injury prevention strategies. Spörri et al. (2012) conducted interviews with representatives from different expert stakeholder groups in alpine skiing World Cup racing. They perceived 32 risk factors concerning four primary categories, namely, athlete, course, equipment and snow. Interviews with experts (Table 4; Spörri et al., 2012) revealed that the top five key risk factors are system ski, plate, binding and boot; changing snow conditions; physical aspects of the athletes; speed and course setting aspects; and speed in general. The injury factors in snowsports can be divided into intrinsic and extrinsic risk factors (van Mechelen et al., 1992; Spörri et al., 2012).

**TABLE 4:** Perceived injury risk factors derived from interviews within the basic categories of athlete, course, equipment, and snow (in alphabetical order).

**Athlete**	**Course**	**Equipment**	**Snow**
Aspects of body temperature	Poor visibility	Binding/plate	Aggressive snow conditions
Athlete’s adaptability	Course maintenance during race	Gates (panels and poles)	Changing snow conditions*
Athlete’s crash behavior	Course setting in general	Protectors and helmets	Smooth snow surface
Athlete’s individual responsibility	Jumps	Racing suits	Techniques in snow preparation
Athlete’s race preparation	Level of course difficulty	Ski	
Fatigue	Safety net position and spill zone	Ski boot	
Genetics and anthropometry	Speed and course setting aspects*	System ski, plate, binding and boot*	
Physical aspects*	Speed and topographic aspects		
Psychological aspects	Speed in general*		
Pre-injury aspects	Topography in general		
Skiing technique and tactics			

* Top five key injury risk factors.

Intrinsic factors include the skill level of athletes, genetic predisposition, sex, and age. Pujol et al. (2007) reported that the incidence of primary ACL injury was higher among alpine skiers who rank in the top 30 in the world (FIS World Rankings). Re-injury and bilateral injury rates were also higher among athletes in the top 30. With regard to unfavorable genetic predisposition, Westin et al. (2016) found that compared with athletes whose parents did not suffer from ACL injuries, the odds ratio to also suffer an ACL injury for those with a parent who suffered from ACL injury was 1.95 (95% CI: 1.04–3.65). Sex has different influences on different snowsports. In alpine skiing, injury incidence and severity were higher in males than in females. However, the incidence of knee injury in any discipline was not different between sexes (Flørenes et al., 2009; Bere et al., 2014). The difference in injury incidence between males and females might be related to different skiing patterns (technique and strategy). A study suggested that men are more prone to take risks than women (Bere et al., 2014). In addition, certain extrinsic factors might have an influence on injury incidence and severity. In alpine skiing World Cup, the course is longer, the vertical drop is higher and skiing speed is faster in men’s events than in women’s events (Bere et al., 2014). Thus, males may have a shorter reaction time than females when facing sudden variations in postural stability and forces acting on the body (Bere et al., 2014). With regard to risk of knee injury, the speed, technical demands and high forces probably overrule any vulnerability factors related to sex (Flørenes et al., 2009). However, no significant difference in injury incidence or knee injury between men and women in snowboarding and freestyle skiing (Torjussen and Bahr, 2006; Flørenes et al., 2010; Major et al., 2014). As noted for alpine skiing, perhaps the technical demands and forces involved in these sports overrule sex factors (Flørenes et al., 2010). With regard to age (biological maturity), in the 2012 Innsbruck YWOG, no significant effect was found on injury risk between athletes aged 14–15 and ≥17 years (Ruedl et al., 2012). However, the majority of the subjects in this study were young athletes. Hence, injury risk in different age groups must be explored. Nevertheless, injuries among young athletes may negatively affect the development of their musculoskeletal structures, potentially resulting in long-term damage (Edouard et al., 2012). This possibility further highlights the importance of preventing injuries in biologically immature athletes.

Extrinsic factors include course setting, discipline and equipment. In alpine skiing, the downhill course is the longest (ranging from 2,000 to 4,500 m) with the highest vertical drop (800 to 1,100 m for males; 450 to 800 m for females) and, consequently, the highest speed (average of 95 to 105 km/h; maximum speed can exceed 140 km/h). These features contribute to high injury incidence in alpine skiing (Flørenes et al., 2009). In freestyle skiing and snowboarding, most injuries occur because of falls and crashes related to jumps, kickers and the halfpipe. In three freestyle skiing World Cup seasons (2006–2009), injury incidence in ski halfpipe and aerials was (18.5–23.9 injuries per 1,000 runs); these are events with challenging aerial maneuvers, and injury incidence in these competitions seems to be at least as high as that in downhill of alpine skiing (Flørenes et al., 2010). Torjussen and Bahr (2006) reported injury risk is 4–8 times higher in big air and halfpipe than in snowboard slalom and giant slalom. In these disciplines, extreme performance, such high jumps and impressive rotations, is the essence of the sport. Technical errors during take-off and landing are the primary cause of injuries (Torjussen and Bahr, 2006; Ruedl et al., 2012). In addition, skis play an important role in injury prevention. The fixation of both feet protects against knee injuries (Sutherland et al., 1996; Rønning et al., 2001) because the ski presumably protects knee ligaments from injuries due to twisting (Pigozzi et al., 1997) and valgus stress (Abu-Laban, 1991). In alpine skiing, skiing speed may be most effectively reduced by increasing the ski–snow friction force in giant slalom and super-G. However, increasing the ski–snow friction force in downhill alpine skiing may be as equally effective as an increase in air drag force (Gilgien et al., 2018). Furthermore, the time and competition schedule are other potential issues. For example, in alpine skiing during the 2012 Innsbruck YWOG, six races were held in eight days, and the athletes often competed in more than one discipline. Physical and mental fatigue may increase injury risk (Ruedl et al., 2012).

## Conclusion

On the basis of injury surveillance studies and data from elite athletes in major snowsport events, this review found that freestyle skiing, alpine skiing, and snowboarding had the highest injury incidence. Injury incidence was higher in snowboard cross and halfpipe, ski halfpipe, aerials, ski cross and downhill alpine skiing. Moreover, the proportion of snowsport injury in competitions and during trainings was similar in general but different in specific events. Furthermore, the knees, shoulders and head were the most commonly and severely injured body parts. The most common injury types were ligament injury, fractures, concussion and muscle and tendon injuries. The most frequent specific diagnosis with high severity was ACL injury. Finally, the main causes of snowsport injuries were collisions, falls and non-contact injuries due to technical error.

Future studies are warranted to explore further the influence of intrinsic and extrinsic risk factors on snowsport injury. Therefore, innovative study designs, such as expert interviews and injury case reconstruction, are essential complementary tools for investigating the biomechanical mechanism of snowsport injuries, identifying disciplines with high risk of injury and providing evidence-based injury prevention measures for all stakeholders in snowsports. Besides, future reviews and investigations should also include recreational winter sports as well as analyze the incidence of injuries in younger age groups.

## Author Contributions

WF and LL: conceptualization, project administration, and funding acquisition. CY and YX: literature retrieval, data curation, and writing—original draft preparation. CY, YX, YY, XZ, SZ, and MZ: literature screening. WF, CY, YX, YY, XZ, SZ, and MZ: review and editing. All authors contributed to the article and approved the submitted version.

## Conflict of Interest

The authors declare that the research was conducted in the absence of any commercial or financial relationships that could be construed as a potential conflict of interest.
